# Barker's Method of Excising the Hip Joint in Tuberculous Disease

**Published:** 1892-01-16

**Authors:** 


### SURGERY.

### BARKER'S METHOD OF EXCISING THE HIP-JOINT IN TUBERCULOUS DISEASE.

In a recent paper on excision in tubercular hip disease we referred to Barker's method of operating, and showed that his results were far superior to those of other operators, and to the non-operative treatment by rest and extension.

We propose now to give the results of Barker's operation, and then to describe his method in detail.

At a meeting of the Medico-Chirurgical Society in November of last year (1890) Barker brought forward a report of seven cases in which he had excised the joint by his method. In all the cases there were signs of abscess about the joint before operation, and disease was found to be present in the acetabulum when the joints were excised. So that they were all fairly advanced cases. Primary union was obtained in six out of seven cases. In the case in which primary union was not obtained, the hip was converted into a large thin walled abscess cavity with thin red skin over it, and the patient was greatly emaciated and debilitated, so that primary union could hardly have been expected. In one case, in which primary union was obtained, there was an intrapelvic abscess with a sequestum in it, communicating with the joint through the perforated acetabulum.

Not only was primary union obtained, but the disease was brought to an end, and after a few months of further rest in double Thomas's splints, the children were able to get about again without splints. Two of the children at the time Mr. Barker brought forward the cases were still in splints; the time had not come for leaving them off.

We have yet to learn the future condition of the limb in these cases, but results such as these—rapid healing of the diseased joint without further suppuration—speak for themselves.

As we mentioned in our former paper, the great principle of Barker's method of excision is so thoroughly to remove the diseased tissues from the joint, that it can be closed up without drainage. Not only does he scrape and cut away all the tubercular material, but as it is detached he washes it out of the wound by constant irrigation with hot water which has been sterilised by being boiled, and finally applies iodoform emulsion to the interior of the joint.

Mr. Pollard has operated on four cases by Barker's method. In all he obtained primary union, but two broke down again, one probably, he thinks, because he made the posterior incision instead of anterior, and could not so well clear all the tuberculous material away; the other because there was a sinus going through the acetabulum. Pollard, however, did not flush the joints as Barker does, but only scraped and sponged them out.

Mr. Deane has also adopted Barker's method, but with this modification—that he drains away the exuded serum for the first twenty-four hours, and he finds that he gets union as satisfactorily without allowing any retention of serum to cause a rise in temperature.

And now we must give some details of the method employed by Barker. Attention to details is of the utmost importance, and to its strict asepsis, as Barker puts it—not so much antisepsis, as asepsis, perfect asepsis of everything coming in contact with the wound, rather than the constant application of an antiseptic and irritating solution to it. The hot water used so freely for washing out the joint, having been sterilised by previous boiling, is a non-irritating aseptic fluid, and its warmth controls oozing during the operation. The water is used at a temperature between 105 and 110, and is supplied in a tin holding two gallons, suspended above the operating table, so as to come with some force along a tube leading from an opening in the bottom of the tin into the instrument used for scraping away all the diseased tissue. This is Barker's flushing gouge, and is made by Khrone and Sesemann. With this instrument the joint is scraped and flushed at the same time. Mr. Barker says that often worthless instruments are made as his flushing gouge, and recommends that the perfected instrument should be procured from the above-named instrument makers.

He always makes the anterior incision of Hueter and Parker, *i.e.*, an incision commencing on the anterior aspect of the thigh, half an inch below the anterior superior spine, and running downwards and a little inwards for three inches, but the deeper cuts need not divide the tissues to the same extent. This anterior incision enables the operator to get at all parts of the joint better than the posterior, and divides no muscles.

If we open an abscess cavity before getting into the joint, it must be thoroughly scraped and flushed out before we proceed further.

The neck of the bone is divided with a saw, and the head removed with the gouge or sequestrum forceps. Then all carious bone and tubercular tissue is scraped away and thoroughly flushed out of the joint so that it cannot infect the freshly cut surfaces in the wound, or remain behind in the joint cavity; and as it is constantly being washed away, we get a clearer view of what remains to be removed by further scraping.

After the tuberculous material has been removed, the joint is thoroughly dried out with carbolised sponges, and one or two left in while the sutures are inserted. These must be of carbolised silk and dip deeply into the tissues. The sponges are removed just before they are tied, and iodoform emulsion is poured into the joint, and allowed to run out as the last stitches are secured. The wound is then dressed with an antiseptic dressing, and needless to say, the parts operated on must have been rendered aseptic before the operation was begun in the usual manner.

But the operation completed, a very important part of the method has yet to be carried out, and that is to make pressure on the joint by means of salicylic wool and a bandage so as to press the surfaces together and get union of the clean scraped walls.

This is Barker's method of excising the hip, and it seems to open up to us a far more hopeful prospect of curing these cases of tuburcular disease than the old method, or the treatment by prolonged rest and drainage. With primary union all the pain and disturbance of dressing the wound is avoided, the chances of septic infection or absorption vanish, and above all we can send the child on a double Thomas's splint at an early date into the country, where the pure invigorating air will help towards complete recovery.

